# Comparison of Agar Dilution to Broth Microdilution for Testing *In Vitro* Activity of Cefiderocol against Gram-Negative Bacilli

**DOI:** 10.1128/JCM.00966-20

**Published:** 2020-12-17

**Authors:** Mariana Albano, Melissa J. Karau, Audrey N. Schuetz, Robin Patel

**Affiliations:** a Division of Clinical Microbiology, Department of Laboratory Medicine and Pathology, Mayo Clinic, Rochester, Minnesota, USA; b Division of Infectious Diseases, Department of Medicine, Mayo Clinic, Rochester, Minnesota, USA; Washington University School of Medicine

**Keywords:** Gram-negative bacilli, *Enterobacterales*, cefiderocol, agar dilution, broth microdilution, Gram-negative bacteria

## Abstract

Cefiderocol (CFDC) is a siderophore cephalosporin with activity against Gram-negative bacterial species that are resistant to carbapenems and other drugs. The MICs of CFDC were determined for 610 Gram-negative bacilli, including 302 multinational *Enterobacterales* isolates with characterized mechanisms of beta-lactam resistance, 180 clinical isolates from the Mayo Clinic and Mayo Clinic Laboratories not characterized for specific resistance mechanisms, and 128 isolates with CFDC MICs of ≥8 μg/ml obtained from International Health Management Associates, Inc. (IHMA, Schaumburg, IL). Broth microdilution using standard cation-adjusted Mueller-Hinton broth (BMD) and iron-depleted cation-adjusted Mueller-Hinton broth (ID-BMD), and agar dilution (AD) using standard Mueller-Hinton agar were performed according to Clinical and Laboratory Standards Institute (CLSI) guidelines. MICs were interpreted according to the investigational CLSI, FDA, and EUCAST breakpoints, and results were compared. MICs inhibiting 50 and 90% of organisms (MIC_50_ and MIC_90_, respectively), essential agreement (EA), categorical agreement (CA), and error of different types were determined. Results showed considerable discordance between AD and ID-BMD. CFDC showed low EA and CA rates and high error rates for AD in comparison to ID-BMD. Overall, this study does not support use of standard AD for determining CFDC MICs.

## Abstract (precis)

Cefiderocol (CFDC) is a siderophore cephalosporin with activity against Gram-negative bacterial species that are resistant to carbapenems and other drugs. The MICs of CFDC were determined for 610 Gram-negative bacilli, including 302 multinational *Enterobacterales* isolates with characterized mechanisms of beta-lactam resistance, 180 clinical isolates from the Mayo Clinic and Mayo Clinic Laboratories not characterized for specific resistance mechanisms, and 128 isolates with CFDC MICs of ≥8 μg/ml obtained from International Health Management Associates, Inc.

## INTRODUCTION

Over the last few decades, infections caused by drug-resistant Gram-negative bacilli (GNB) have become a public health problem worldwide because of their prevalence and the proliferation of resistance mechanisms (1, 2). According to the U.S. Centers for Disease Control and Prevention's 2019 Antibiotic Resistance Threats report, more than 2.8 million antibiotic-resistant infections occur in the United States each year, resulting in almost 36,000 deaths (3). The report describes three categories of threat based on level of concern for human health: urgent, serious, and concerning. Carbapenem-resistant Acinetobacter spp. and *Enterobacterales* are considered urgent threats, with extended-spectrum beta-lactamase (ESBL)-producing *Enterobacterales* and multidrug-resistant Pseudomonas aeruginosa being considered serious threats.

Of particular challenge are the multidrug-resistant (MDR) *Enterobacterales*, which have developed β-lactam resistance via (i) production of β-lactamases, including ESBLs, carbapenemases, and plasmid-mediated AmpC (4, 5); (ii) mutations in outer membrane porins, leading to loss of porins, decreased porin expression, or porins with narrow channels; and/or (iii) upregulation of efflux pumps, which pump antibiotics out of bacterial cells (6, 7).

Although several β-lactam–β-lactamase inhibitor antibiotic combinations have been or are in development, they are not active against all classes of β-lactamases. One way to avoid or, at least, reduce the effect of β-lactamases in GNB is to ensure that the antimicrobial is able to access the periplasmic space (8). A strategy to accomplish this is to leverage an essential element—iron—using siderophores to bind to it. Antibiotics can be attached to siderophores, creating a complex that is recognized by specific bacterial iron uptake systems. The antibiotic can then be released into the periplasmic space, in what has been referred to as a Trojan horse tactic (8, 9).

Cefiderocol (CFDC) is a novel siderophore cephalosporin which is actively transported into the periplasmic space along with ferric iron and binds mainly to penicillin-binding protein 3 (PBP3) of GNB, inhibiting bacterial cell wall synthesis (10). CFDC is broadly stable against hydrolysis by class A, B, C, and D β-lactamases, including carbapenemases and ESBLs (11–13), being one of the first U.S. Food and Drug Administration (FDA)-approved/cleared agents with activity against Ambler class B β-lactamases, including New Delhi metallo-β-lactamase (NDM), imipenemase (IMP), and Verona integron-encoded metallo-β-lactamase (VIM). CFDC has been approved by the FDA for treatment of complicated urinary tract infections (14), and a phase III clinical trial for treatment of nosocomial pneumonia, including health care-associated pneumonia and hospital/ventilator-associated pneumonia, has been completed (15). In Europe, CFDC has also received marketing authorization (16).

Although the gold standard susceptibility test for CFDC is broth microdilution (BMD), agar dilution (AD) is considered a reliable and less expensive method for many other antibiotics and is in routine use in some laboratories. Agar dilution is particularly useful for batch testing. Published studies evaluating the activity of CFDC against GNB have focused on BMD. Here, we evaluated AD in comparison to BMD (with and without iron-depleted medium) for testing the *in vitro* antimicrobial activity of CFDC against a diverse collection of GNB, including subsets enriched for CFDC-resistant and drug-resistant organisms.

## MATERIALS AND METHODS

### Bacterial isolates.

**FIG 1:**
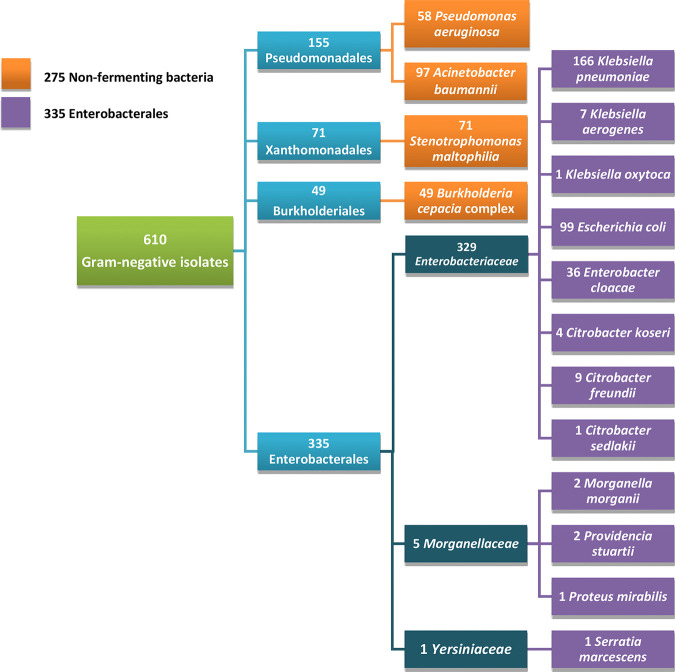
The 610 Gram-negative bacillus isolates tested, including 275 nonfermenting bacteria and 335 *Enterobacterales*.

A collection of 610 GNB was studied (Fig. 1). Of these, 302 were multinational *Enterobacterales* isolates previously collected from the United States, Canada, and Singapore (17–24), possessing one or more of the following genotypic resistance mechanisms as determined by β-lactamase gene-specific PCR: *bla*_CMY_, *bla*_CTX-M_, *bla*_FOX_, *bla*_IMI_, *bla*_IMP_, *bla*_KPC_, *bla*_NDM_, *bla*_OXA-48-like_, *bla*_SHV_, *bla*_SME_, and *bla*_TEM_. These isolates included 155 Klebsiella pneumoniae, 99 Escherichia coli, 21 Enterobacter cloacae, 8 Citrobacter freundii, 5 Serratia marcescens, 4 Citrobacter koseri, 3 Klebsiella aerogenes, 2 Morganella morganii, and 2 Providencia stuartii isolates and 1 Citrobacter sedlakii, 1 Klebsiella oxytoca, and 1 Proteus mirabilis isolate; 128 were isolates obtained from International Health Management Associates, Inc. (IHMA, Schaumburg, IL), with CFDC MICs of ≥8 μg/ml (13, 25), including 91 Acinetobacter baumannii, 15 E. cloacae, 11 K. pneumoniae, 4 K. aerogenes, 3 P. aeruginosa, 2 S. marcescens, 1 Burkholderia cepacia, and 1 C. freundii isolate; and180 were isolates from the Mayo Clinic and Mayo Clinic Laboratories that were not genotypically characterized, including 55 P. aeruginosa, 71 Stenotrophomonas maltophilia, 48 Burkholderia cepacia complex, and 6 A. baumannii isolates. All isolates had been stored at −80°C in MicroBank vials (Pro-Lab Diagnostics, Round Rock, TX) prior to testing.

### Broth microdilution.

Premade frozen panels prepared by IHMA were used to determine MICs of CFDC by BMD using cation-adjusted Mueller-Hinton broth (CAMHB), and iron-depleted cation-adjusted Mueller-Hinton broth (ID-CAMHB) (≤0.03 mg/liter iron), following Clinical and Laboratory Standards Institute (CLSI) guidelines (26, 27). In brief, 100 μl of an ∼10^8^-CFU/ml suspension of bacteria was diluted in 2.9 ml of sterile water, and 10 μl was inoculated into each well, resulting in ∼5 × 10^4^ CFU/well; CFDC concentrations were studied in 2-fold dilutions ranging from 0.004 to 64 μg/ml. Panels used for testing *Enterobacterales* and P. aeruginosa were incubated for 16 to 20 h and 20 to 24 h for testing A. baumannii, S. maltophilia, and B. cepacia complex, at 37°C. E. coli ATCC 25922 and P. aeruginosa ATCC 27853 were used as quality control (QC) strains and included in each trial. Panels were read according to CLSI guidelines, with MICs determined as concentrations in wells in which growth was significantly reduced, ignoring tiny buttons or light or faint turbidity (26).

### Agar dilution.

MICs were determined by AD using Mueller-Hinton agar (MHA) (BD Difco) plates prepared with 2-fold dilutions of CFDC ranging from 0.004 to 64 μg/ml in accordance with CLSI guidelines, although there is no specific recommendation to test CFDC by AD (26, 27). In brief, CFDC was prepared and diluted in MHA according to CLSI guidelines, and 10 ml was placed in sterile petri dishes. Plates were prepared fresh daily. Colonies from blood agar plates were suspended in saline (∼10^8^ CFU/ml), and 300 μl was added to 2.7 ml of saline. Agar plates were inoculated using a multi-inoculator (Steers replicator), resulting in a bacterial density of ∼10^4^ CFU/spot. Plates testing *Enterobacterales* and P. aeruginosa were incubated for 16 to 20 h, and those testing A. baumannii, S. maltophilia, and B. cepacia complex were incubated for 20 to 24 h at 37°C. E. coli ATCC 25922 and P. aeruginosa ATCC 27853 were used as QC strains in each trial. The MIC was reported as the lowest concentration with no visible growth, a single colony, or a faint haze due to the inoculum (27).

### Determination of ion concentration in MHA.

The fluid phase of hydrated MHA was prepared according to the method described by Hawkey et al. (28). Melted 40-ml aliquots were frozen at −80°C and then thawed in a water bath at 80°C. The process was repeated, and MHA was centrifuged at 47,000 × *g* for 10 min to pellet insoluble components of the medium. The clear supernatant was decanted, and the amount of iron was determined using an iron test kit according to the manufacturer’s instructions (Visocolor HE Iron; Macherey-Nagel, Germany). Testing was performed twice.

### Data analysis.

The MICs required to inhibit 50 and 90% of organisms (MIC_50_ and MIC_90_, respectively) were calculated. Essential agreement (EA) was assessed by calculating the percentage of isolates with MICs within 1 doubling dilution of that determined by BMD with ID-CAMHB (ID-BMD). Categorical agreement (CA) was assessed by calculating the percentage of isolates tested by AD that yielded the same categories as ID-BMD. A percentage of ≥90% was considered acceptable for EA and CA. Categorical results that were not congruent were categorized as follows: minor error (mE), major error (ME), and very major error (VME). Acceptable percentages of errors were ≤1.5% for VME, ≤3% for ME, and ≤7% for combined mE and ME (29). CLSI, the FDA, and the European Committee on Antimicrobial Susceptibility Testing (EUCAST) have different breakpoints for CFDC determined by ID-BMD (Table 1); no breakpoints are defined for AD (16, 26, 30). Here, MICs of A. baumannii and S. maltophilia were interpreted according to investigational CLSI breakpoints (since there are no FDA or EUCAST breakpoints), and MICs of P. aeruginosa and *Enterobacterales*, according to FDA breakpoints. Because there are no CLSI, FDA or EUCAST breakpoints established for B. cepacia complex, results for species in this complex were reported as MIC_50_ and MIC_90_ only.

**TABLE 1:** FDA, CLSI, and EUCAST breakpoint values applied for each group or species^*a*^

Organism	MIC breakpoint (μg/ml)^*a*^								
	CLSI (investigational)			FDA			EUCAST		
	S	I	R	S	I	R	S	I	R
Pseudomonas aeruginosa	≤4	8	≥16	≤1	2	≥4	≤2		>2
Acinetobacter baumannii and Stenotrophomonas maltophilia	≤4	8	≥16	NA	NA	NA	NA	NA	NA
*Enterobacterales*	≤4	8	≥16	≤2	4	≥8	≤2		>2

a S, susceptible; I, intermediate; R, resistant; NA, not applicable.

## RESULTS

**FIG 2:**
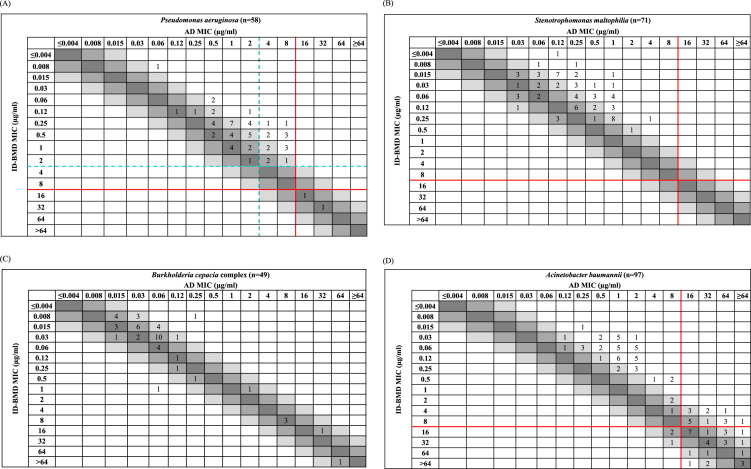

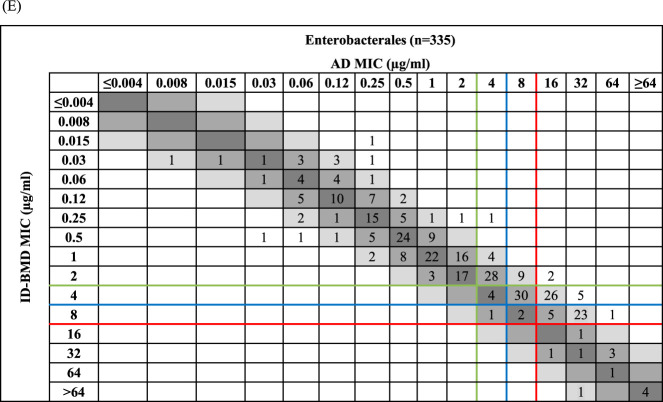
Scattergrams of cefiderocol MICs obtained by agar dilution (AD) versus broth microdilution using iron-depleted Mueller-Hinton agar (ID-BMD) for Pseudomonas aeruginosa (A), Stenotrophomonas maltophilia (B), Burkholderia cepacia complex (C), Acinetobacter baumannii (D), and *Enterobacterales* (E). Lines represent the applied resistance breakpoints: red, investigational CLSI breakpoints (26); blue, FDA breakpoints (29); green, EUCAST breakpoints (16); blue-green dashed line, overlapping EUCAST and FDA breakpoints.

The *in vitro* activity of CFDC as assessed by the different methods is summarized in Table 2, with analyses of each bacterial group by CLSI, FDA, and EUCAST breakpoints. For the 610 isolates tested, regardless of species, MICs obtained by BMD with standard CAMHB were, as expected, higher than those obtained by ID-BMD (Table S1), so we focused on comparison between AD and ID-BMD. Scattergrams showing MICs for ID-BMD and AD for P. aeruginosa, S. maltophilia, B. cepacia complex, A. baumannii and *Enterobacterales* are shown in Fig. 2; cumulative percentages of isolates of all groups and species inhibited at each CFDC concentration tested are shown in Fig. S1; and QC MICs for each experiment are shown in Table S2.

**TABLE 2:** MICs and MIC interpretation for cefiderocol according to CLSI (26), FDA (29), and EUCAST (16) breakpoints for Gram-negative bacilli (*n* = 610)

Organism (no. of isolates)	Method	MIC^*a*^ (μg/ml)			No. (%) with MIC interpretation							
		Range	MIC_50_	MIC_90_	Susceptible			Intermediate		Resistant		
					CLSI^*b*^	FDA^*c*^	EUCAST^*d*^	CLSI	FDA	CLSI	FDA	EUCAST
Pseudomonas aeruginosa (58)	ID-CAMHB	0.008–32	0.5	2	56 (97)	52 (89)	56 (97)	0	4 (7)	2 (3)	2 (3)	2 (3)
	AD	0.06–32	2	8	48 (83)	27 (48)	41 (70)	8 (14)	13 (22)	2 (3)	17 (30)	17 (30)
Stenotrophomonas maltophilia (71)	ID-CAMHB	0.004–0.5	0.06	0.25	71 (100)	NA^*e*^	NA	0	NA	0	NA	NA
	AD	0.015–4	0.25	1	71 (100)	NA	NA	0	NA	0	NA	NA
Burkholderia cepacia complex (49)	ID-CAMHB	0.008–>64	0.03	1	NA	NA	NA	NA	NA	NA	NA	NA
	AD	0.015–64	0.06	1	NA	NA	NA	NA	NA	NA	NA	NA
Acinetobacter baumannii (97)	ID-CAMHB	0.015–>64	2	32	55 (57)	NA	NA	10 (10)	NA	32 (33)	NA	NA
	AD	0.125–>64	8	64	44 (45)	NA	NA	8 (8)	NA	45 (46)	NA	NA
*Enterobacterales* (335)^*f*^	ID-CAMHB	0.015–>64	2	8	288 (86)	223 (67)	223 (67)	32 (9)	65 (19)	15 (5)	47 (14)	112 (33)
	AD	0.008–>64	2	32	217 (65)	178 (53)	179 (53)	41 (12)	40 (12)	77 (23)	117 (35)	156 (47)
Klebsiella pneumoniae (166)^*f*^	ID-CAMHB	0.03–>64	2	8	146 (88)	103 (62)	103 (62)	15 (9)	44 (26)	5 (3)	20 (12)	63 (38)
	AD	0.03–>64	2	32	96 (58)	81 (48)	81 (48)	19 (11)	16 (9)	51 (31)	69 (42)	85 (51)
Escherichia coli (99)^*f*^	ID-CAMHB	0.03–>64	1	4	94 (95)	82 (83)	82 (83)	3 (3)	12 (12)	2 (2)	5 (5)	17 (17)
	AD	0.008–>64	1	8	83 (84)	69 (70)	69 (70)	8 (8)	14 (14)	8 (8)	16 (16)	30 (30)
Enterobacter cloacae (36)^*f*^	ID-CAMHB	0.25–32	4	16	20 (55)	17 (47)	17 (47)	10 (28)	3 (9)	6 (17)	16 (44)	19 (53)
	AD	0.06–64	4	32	18 (50)	13 (36)	13 (36)	4 (11)	5 (14)	14 (39)	18 (50)	23 (64)
Other *Enterobacterales* (34)^*f*^	ID-CAMHB	0.015–64	1	8	28 (82)	22 (64)	22 (64)	4 (12)	6 (18)	2 (6)	6 (18)	12 (36)
	AD	0.03–64	4	32	21 (62)	16 (47)	16 (47)	6 (18)	5 (15)	7 (21)	13 (38)	18 (53)

a MIC_50_ and MIC_90_ were calculated for each genus or species with >20 isolates tested.

b Investigational CLSI breakpoints for S. maltophilia, A. baumannii and *Enterobacterales*: susceptible (S), ≤4 μg/ml; intermediate (I), 8 μg/ml; resistant (R), ≥16 μg/ml.

c FDA breakpoints for P. aeruginosa: S, ≤1 μg/ml; I, 2 μg/ml; R, ≥4 μg/ml; for *Enterobacterales*: S, ≤2 μg/ml; I, 4 μg/ml; R, ≥8 μg/ml. The FDA breakpoints for the *Enterobacterales* (listed as *Enterobacteriaceae* on the FDA website) are specific for E. coli, K. pneumoniae, P. mirabilis, and E. cloacae complex (30).

d EUCAST breakpoints for P. aeruginosa and *Enterobacterales*: S, ≤2 μg/ml; R, >2 μg/ml.

e NA, no breakpoints available.

f The 335 isolates of *Enterobacterales* were composed of 166 K. pneumoniae, 99 E. coli, 36 E. cloacae, 9 C. freundii, 7 S. marcescens, 4 *C. koseri*, 7 K. aerogenes, 2 M. morganii, and 2 P. stuartii isolates and 1 P. mirabilis, 1 *C. sedlakii*, and 1 K. oxytoca isolate. Species of *Enterobacterales* with <20 isolates were grouped in “other *Enterobacterales*.”

By AD, 83, 48, and 70% of P. aeruginosa isolates were susceptible to CFDC using investigational CLSI, FDA, and EUCAST breakpoints, respectively; by ID-BMD, 97, 89, and 97%, respectively, were susceptible to CFDC. When AD was compared to ID-BMD, there was 38% EA, with 86, 52, and 74% CA when investigational CLSI, FDA, and EUCAST breakpoints were applied, respectively. All susceptible isolates were isolates from the Mayo Clinic or Mayo Clinic Laboratories which were not specifically characterized genetically.

All 71 S. maltophilia isolates tested (from the Mayo Clinic and Mayo Clinic Laboratories) had MICs of ≤4 μg/ml (investigational CLSI susceptible breakpoint), with MIC_90_ values of 1 and 0.25 μg/ml for AD and ID-BMD, respectively. There was 100% CA and 30% EA for AD versus ID-BMD.

B. cepacia complex isolates had MIC_50_ and MIC_90_ values of 0.06 and 1 μg/ml by AD, and of 0.03 and 1 μg/ml by ID-BMD, with 77% EA for AD versus ID-BMD. CA and error rates could not be calculated due to the absence of breakpoints for this species complex.

A. baumannii isolates were 45% (44/97) and 57% (55/97) susceptible to CFDC by AD and ID-BMD, respectively, according to the investigational CLSI breakpoints; EA and CA of AD versus ID-BMD were 32 and 76%, respectively, with 6% ME and 16% mE. No FDA and EUCAST breakpoints have been established for this species.

At ≤2 μg/ml (FDA and EUCAST susceptible breakpoints), CFDC inhibited 53% and 67% of *Enterobacterales* isolates when MICs were determined with AD and ID-BMD, respectively (Table 2). MIC_50_ and MIC_90_ values determined by AD were 2 and 32 μg/ml, and those determined by ID-BMD were 2 and 8 μg/ml. Comparing AD versus ID-BMD, there was 62% EA and 61% CA, with 25% mE and 13% ME, applying FDA breakpoints (Table 3).

**TABLE 3:** Agreement and errors for AD compared with ID-BMD obtained applying CLSI (26), FDA (29), and EUCAST (16) breakpoints

Organism (no. of isolates)	% EA^*a*^ (±1 doubling dilution)	% CA			No. (%) of errors								
					mE			ME			VME		
		CLSI	FDA	EUCAST	CLSI	FDA	EUCAST	CLSI	FDA	EUCAST	CLSI	FDA	EUCAST
Pseudomonas aeruginosa (58)	38	86	52	74	8 (14)	16 (27)	NA	0	12 (20)	15 (26)	0	0	0
Stenotrophomonas maltophilia (71)	30	100	NA	NA	0	NA	NA	0	NA	NA	0	NA	NA
Burkholderia cepacia complex (49)	77	NA	NA	NA	NA	NA	NA	NA	NA	NA	NA	NA	NA
Acinetobacter baumannii (97)	32	76	NA	NA	16 (16)	NA	NA	6 (6)	NA	NA	0	NA	NA
*Enterobacterales* (335)	62	69	61	72	62 (18)	84 (25)	NA	40 (12)	44 (13)	67 (20)	0	0	24 (7)
* *Klebsiella pneumoniae (166)	57	60	47	57	31 (19)	48 (29)	NA	35 (21)	39 (23)	46 (28)	0	0	24 (14)
* *Escherichia coli (99)	78	86	84	87	13 (13)	23 (23)	NA	3 (3)	2 (2)	13 (13)	0	0	0
* *Enterobacter cloacae (36)	73	67	78	89	12 (33)	8 (22)	NA	0	0	4 (11)	0	0	0
* *Citrobacter freundii (9)	44	89	67	33	1 (11)	2 (22)	NA	0	1 (11)	3 (33)	0	0	0
* *Serratia marcescens (7)	71	71	71	86	1 (14)	2 (28)	NA	1 (14)	0	1 (14)	0	0	0
* *Klebsiella aerogenes (7)	29	14	43	86	5 (71)	4 (57)	NA	1 (14)	0	1 (14)	0	0	0
* *Citrobacter koseri (4)	75	100	100	100	0	0	NA	0	0	0	0	0	0
Other species^*b*^ (7)	100	86	71	86	1 (14)	2 (28)	NA	0	0	1 (14)	0	0	0

a EA, essential agreement (acceptability criterion, ≥90%); CA, categorical agreement (acceptability criterion, ≥90%); mE, minor error; ME, major error (acceptability criterion, ≤3%); VME, very major error (acceptability criterion, ≤1.5%); NA, not applicable.

b 2 M. morganii, 2 P. stuartii, 1 P. mirabilis, 1 *C. sedlakii*, and 1 K. oxytoca isolate.

Using AD, E. coli, K. pneumoniae, and E. cloacae were 70, 48, and 36% susceptible to CFDC when analyzed by FDA breakpoints; when results were obtained using ID-BMD, susceptibility rates were 83, 62, and 47%, respectively, with EA rates of 78, 57, and 73% and CA rates of 84, 47, and 78% when AD was compared to ID-BMD, and with 23, 29, and 22% mE and 2, 23, and 0% ME, respectively.

EA rates for *Enterobacterales* isolates harboring *bla*_CTX-M_, *bla*_NDM_, and *bla*_KPC_ were 87, 67, and 63%, respectively, when AD was compared to ID-BMD; an acceptability criterion of ≥90% was not met in any group. CA rates applying investigational CLSI and FDA breakpoints were 93 and 85%, respectively, for *bla*_CTX-M_-carrying isolates, 53 and 56%, respectively, for *bla*_NDM_-carrying isolates, and 63 and 61%, respectively, for *bla*_KPC_-carrying isolates.

The amount of iron determined in the MHA lot used in this study was >0.2 μg/ml.

## DISCUSSION

Iron is an essential nutrient for both humans and pathogens. During infection in mammalian hosts, the innate immune system limits iron availability by hijacking iron to deprive pathogens of this essential nutrient (31). Therefore, low availability of iron *in vivo* during infection can be likened to iron depletion in broth *in vitro*. Depletion of iron in BMD media for CFDC susceptibility testing has been previously demonstrated to recapitulate *in vivo* activity of CFDC (32), and therefore, this is the method approved by CLSI (26, 33). Our results, like those of others (25, 34), support the use of iron-depleted media on the basis of the higher MICs obtained when CFDC was tested with standard CAMHB (>0.03 μg/ml iron) than with iron-depleted CAMHB (≤0.03 μg/ml iron; Table S1) (35).

To the best of our knowledge, there are no published studies evaluating AD testing of CFDC. We showed low EA values (not meeting a 90% acceptance level) for all species tested, in addition to poor CA (except for S. maltophilia isolates, although all study isolates had low MICs), and high rates of mE and ME, with some VME when results of AD were compared to those of ID-BMD. Although the medium used for AD itself has been reported to have iron-chelating properties (36) and lower free-iron concentrations than CAMHB (37), the identified discordance between AD and ID-BMD could possibly have been related to the amount of iron in the MHA lot used for the duration of the study, as it was at least 6 times more than the maximum amount allowed in BMD media (0.03 μg/ml).

MICs may be influenced by cation concentration of the culture medium, as demonstrated by Washington et al., who assessed activity of aminoglycosides against P. aeruginosa isolates using 14 lots of MHA (38). Girardello et al. demonstrated variability in polymyxin B MICs determined by AD in comparison to BMD using four MHA brands (37). MICs of tigecycline determined by Etest were 2 to 8 times higher with different MHA commercial brands and appeared to depend on the concentration of manganese (39). Thus, the amount of iron present in the specific MHA medium used for AD here may have affected CFDC activity.

It is noteworthy that all 71 S. maltophilia isolates tested were susceptible to CFDC, with MIC_90_ values of 1 and 0.25 μg/ml obtained by AD and ID-BMD, respectively. MIC_90_ values reported in previous surveillance studies ranged from 0.25 to 0.5 μg/ml for ID-BMD (13, 25). The activity of CFDC against S. maltophilia may be significant, given that this species is intrinsically resistant to many broad-spectrum antimicrobial agents, including carbapenems, as a result of production of inducible chromosomal metallo- and serine-β-lactamases (L1 and L2) (40). The S. maltophilia and B. cepacia complex findings suggest that CFDC deserves further study for these often challenging-to-treat bacterial species.

We evaluated discrepancies between categorization by MIC breakpoints defined by the FDA, CLSI, and EUCAST—most were observed among AD results. The investigational CLSI breakpoints are for research use as well as compassionate use of the agent when there is no other therapy available. In 2019, the FDA set breakpoints for CFDC that are more conservative than those of the CLSI (Table 1); FDA breakpoints should be used by laboratories until CLSI reevaluates its investigational breakpoints based on outcomes from more recent clinical trials. A possible revision is expected in 2021 (9). In May 2020, EUCAST established clinical breakpoints for *Enterobacterales* and P. aeruginosa (16).

In this study, we applied FDA breakpoints for all *Enterobacterales*, although the recommendation is technically only for some *Enterobacteriaceae*, including E. coli, K. pneumoniae, E. cloacae complex, and P. mirabilis. For *Enterobacterales*, the susceptibility percentages obtained with ID-BMD were 65, 53, and 53% when investigational CLSI, FDA, and EUCAST breakpoints, respectively, were applied (Table 2). FDA and EUCAST have the same susceptible breakpoints for *Enterobacterales*. For P. aeruginosa, 96 and 89% of study isolates would be considered susceptible using investigational CLSI and FDA breakpoints, respectively. Discrepancies between MIC breakpoints also affected CA and error rates (Table 3). CA for P. aeruginosa when AD was compared to ID-BMD was 86, 52, and 74% when analyzed by investigational CLSI, FDA, and EUCAST breakpoints, respectively; there were 16 mEs when FDA breakpoints were applied and 8 when investigational CLSI breakpoints were applied.

Limitations of this study must be considered. AD using iron-depleted medium was not assessed. The collection of isolates studied was not representative of isolates in general clinical practice, nor was this a “surveillance” study. Instead, this study was enriched with a subset of drug-resistant GNB and included 83 CFDC-resistant isolates representing various species to challenge the breakpoints for method comparison purposes. A large number of A. baumannii isolates were specifically included on the basis of their being CFDC resistant. The main aim of this study was to compare the performance of AD to ID-BMD for MIC determination; therefore, we desired to test a range of susceptible to resistant CFDC isolates. Another limitation was the lack of genetic data regarding mechanisms of resistance for the nonfermenting GNB studied. Also, only a single lot of MHA was evaluated (BD Difco); differences in the cation composition of the MHA can generate categorical errors in susceptibility testing. On the other hand, inclusion of MDR GNB isolates from different countries representing a variety of MICs across the susceptible-resistant spectrum and mechanisms of resistance can be considered a strength of our work.

Overall, CFDC showed low EA rates and high error rates with AD in comparison to ID-BMD. The activity of CFDC against S. maltophilia alongside B. cepacia complex is encouraging. Based on the findings of this study, AD should not be used for *in vitro* susceptibility testing of CFDC using the described method.

## Supplementary Material

Supplemental file 1
