# Supplementary material for: Comparison of Agar Dilution to Broth Microdilution for Testing In Vitro Activity of Cefiderocol against Gram-Negative Bacilli
Source: J Clin Microbiol. 2020 Dec 17;59(1):e00966-20. doi: 10.1128/JCM.00966-20 (PMC7771473; doi:10.1128/JCM.00966-20)
Supplement: Supplemental file 1 [file JCM.00966-20-s0001.pdf]

Table S1. Minimum inhibitory concentration (MIC) of cefiderocol for 610 Gram-negative bacilli determined by broth microdilution using iron-depleted cation-adjusted Muller Hinton broth (ID-BMD), broth microdilution using standard cation-adjusted Muller Hinton broth (BMD), and agar dilution (AD).

**Species or complex**	**Isolate number**	**Resistance gene(s) detected**	**MIC (µg/mL)**		
			**ID-BMD**	**BMD**	**AD**
Escherichia coli	IDRL-10366	bla_{KPC}	1	2	2
E. coli	IDRL-10402	bla_{KPC}	2	4	2
E. coli	IDRL-10407	bla_{NDM}	2	8	4
E. coli	IDRL-10414	$bla_{\text{TEM-12}}$	0.12	0.12	0.12
E. coli	IDRL-10418	$bla_{\text{CTX-M-15}}$, $bla_{\text{TEM-1}}$	0.12	0.25	0.25
E. coli	IDRL-10419	$bla_{\text{CTX-M-15}}$, $bla_{\text{OXA-1}}$	0.12	0.06	0.06
E. coli	IDRL-10424	$bla_{\text{CMY-2}}$	0.25	0.5	0.5
E. coli	IDRL-10430	$bla_{\text{CMY-2}}$	1	1	1
E. coli	IDRL-10431	$bla_{\text{FOX-5}}$	0.06	0.06	0.06
E. coli	IDRL-10432	$bla_{\text{CTX-M-14}}$, $bla_{\text{TEM-1}}$	0.5	0.06	0.12
E. coli	IDRL-10433	$bla_{\text{CTX-M-15}}$, $bla_{\text{TEM-1}}$	1	4	2
E. coli	IDRL-10435	$bla_{\text{CMY-2}}$	0.5	0.25	0.25
E. coli	IDRL-10438	$bla_{\text{CTX-M-14}}$, $bla_{\text{TEM-1}}$	0.5	0.25	0.5
E. coli	IDRL-10439	$bla_{\text{CTX-M-15}}$, $bla_{\text{OXA-1}}$	4	8	4
E. coli	IDRL-10440	$bla_{\text{CMY-2}}$	0.12	0.06	0.06
E. coli	IDRL-10452	$bla_{\text{NDM-1}}$	8	>64	32
E. coli	IDRL-10455	$bla_{\text{NDM-1}}$	4	16	8
E. coli	IDRL-10456	$bla_{\text{NDM-1}}$	4	32	16
E. coli	IDRL-10458	$bla_{\text{NDM-1}}$	4	16	8
E. coli	IDRL-10466	$bla_{\text{NDM-7}}$	4	32	16
E. coli	IDRL-10467	$bla_{\text{NDM-1}}$	4	32	16
E. coli	IDRL-10469	$bla_{\text{OXA-48}}$	0.12	0.06	0.12
E. coli	IDRL-10483	bla_{IMP}	0.06	0.06	0.06
E. coli	IDRL-10503	bla_{KPC}	2	4	2
E. coli	IDRL-10516	bla_{NDM}	4	8	4
E. coli	IDRL-10525	bla_{KPC}	4	8	4
E. coli	IDRL-10553	bla_{KPC}	1	1	1
E. coli	IDRL-10590	$bla_{\text{CMY-8}}$	2	4	2
E. coli	IDRL-10591	$bla_{\text{CTX-M-15}}$	0.12	0.12	0.12
E. coli	IDRL-10592	$bla_{\text{CTX-M-128}}$	1	1	1
E. coli	IDRL-10593	$bla_{\text{CTX-M-15}}$	2	2	2
E. coli	IDRL-10594	$bla_{\text{TEM-43}}$	>64	>64	32
E. coli	IDRL-10595	$bla_{\text{TEM-12}}$	2	4	4
E. coli	IDRL-10596	$bla_{\text{CTX-M-9}}$, $bla_{\text{TEM-1}}$	0.12	0.12	0.12
E. coli	IDRL-10597	$bla_{\text{TEM-1}}$, $bla_{\text{SHV-1}}$ (non-ESBL)	1	2	2
E. coli	IDRL-10598	bla_{SHV}, $bla_{\text{CTX-M-9}}$	2	8	4
E. coli	IDRL-10603	$bla_{\text{CMY-2}}$	0.03	0.06	0.03
E. coli	IDRL-10604	$bla_{\text{CMY-2}}$	0.5	0.5	0.5
E. coli	IDRL-10605	$bla_{\text{CMY-2}}$, $bla_{\text{TEM-1}}$	0.5	1	1
E. coli	IDRL-10606	$bla_{\text{CMY-2}}$, $bla_{\text{TEM-1}}$	0.06	0.12	0.12
E. coli	IDRL-10607	$bla_{\text{CMY-2}}$, $bla_{\text{TEM-1}}$	2	1	1
E. coli	IDRL-10608	$bla_{\text{CTX-M-28}}$	0.06	0.12	0.12
E. coli	IDRL-10633	$bla_{\text{CTX-M-9}}$, $bla_{\text{TEM-1}}$	0.03	0.12	0.12
E. coli	IDRL-10635	$bla_{\text{CMY-2}}$	0.5	0.5	0.5
E. coli	IDRL-10637	$bla_{\text{CTX-M-15}}$, $bla_{\text{TEM-1/84/103/104/122}}$	0.12	0.06	0.06
E. coli	IDRL-10638	$bla_{\text{CTX-M-24}}$, $bla_{\text{TEM-1/84/103/104/122}}$	0.03	0.008	0.008
E. coli	IDRL-10640	$bla_{\text{SHV-1}}$, $bla_{\text{CMY-2}}$	1	0.5	1
E. coli	IDRL-10641	$bla_{\text{CMY-2}}$	2	8	4
E. coli	IDRL-10642	$bla_{\text{CMY-2}}$	1	1	1
E. coli	IDRL-10643	$bla_{\text{CTX-M1-LIKE}}$, $bla_{\text{CMY-2}}$	2	2	2
E. coli	IDRL-10644	$bla_{\text{CTX-M1-LIKE}}$, $bla_{\text{CMY-2}}$	2	8	4
E. coli	IDRL-10645	$bla_{\text{CTX-M-1}}$, $bla_{\text{TEM-1}}$	0.25	0.25	0.25
E. coli	IDRL-10646	$bla_{\text{CMY-2}}$	0.5	0.5	0.5
E. coli	IDRL-10650	$bla_{\text{CTX-M-28}}$	0.5	0.5	0.5
E. coli	IDRL-10651	$bla_{\text{TEM-1}}$, bla_{SHV}, $bla_{\text{CMY-2}}$	0.5	0.5	0.5
E. coli	IDRL-10653	$bla_{\text{CMY-2}}$	0.5	0.5	0.5
E. coli	IDRL-10655	$bla_{\text{TEM-1}}$, bla_{SHV}, $bla_{\text{CTX-M-24}}$, $bla_{\text{CMY-2}}$	0.25	0.5	0.5
E. coli	IDRL-10657	$bla_{\text{CTX-M-1-LIKE}}$, $bla_{\text{TEM-1}}$	2	8	4
E. coli	IDRL-10659	$bla_{\text{CMY-2}}$, $bla_{\text{TEM-1}}$	0.12	0.06	0.06
E. coli	IDRL-10668	bla_{TEM}, bla_{SHV}	2	16	8
E. coli	IDRL-10669	$bla_{\text{TEM-3}}$	0.12	0.12	0.12
E. coli	IDRL-10683	$bla_{\text{CTX-M-15}}$	2	2	2
E. coli	IDRL-10684	$bla_{\text{CTX-M-15}}$	2	16	8
E. coli	IDRL-10685	$bla_{\text{CTX-M-15}}$	0.06	0.25	0.25
E. coli	IDRL-10686	$bla_{\text{CTX-M-15}}$	0.5	1	1
E. coli	IDRL-10687	$bla_{\text{CTX-M-3}}$	0.5	0.5	0.5
E. coli	IDRL-10688	$bla_{\text{CTX-M-3}}$	0.25	0.25	0.25
E. coli	IDRL-10689	$bla_{\text{CTX-M-3}}$	0.25	0.25	0.25
E. coli	IDRL-10705	$bla_{\text{CTX-M-15}}$	0.03	0.03	0.015
E. coli	IDRL-10706	$bla_{\text{CTX-M-15}}$	1	2	2
E. coli	IDRL-10707	$bla_{\text{CTX-M-15}}$	2	4	4
E. coli	IDRL-10708	$bla_{\text{CTX-M-15}}$	4	16	8
E. coli	IDRL-10709	$bla_{\text{CTX-M-15}}$	2	4	4
E. coli	IDRL-10710	$bla_{\text{CTX-M-15}}$	0.5	0.25	0.5
E. coli	IDRL-10711	$bla_{\text{CTX-M-15}}$	2	2	2
E. coli	IDRL-10712	$bla_{\text{CTX-M-15}}$	0.5	0.5	1
E. coli	IDRL-10713	$bla_{\text{CTX-M-15}}$	1	1	1
E. coli	IDRL-10714	$bla_{\text{CTX-M-15}}$	1	0.5	1
E. coli	IDRL-10715	$bla_{\text{CTX-M-15}}$	2	2	2
E. coli	IDRL-10716	$bla_{\text{CTX-M-15}}$	2	2	2
E. coli	IDRL-10719	$bla_{\text{CTX-M-15}}$	0.25	0.25	0.25
E. coli	IDRL-10720	$bla_{\text{CTX-M-15}}$	0.12	0.12	0.12
E. coli	IDRL-10721	$bla_{\text{CTX-M-15}}$	2	4	4
E. coli	IDRL-10722	$bla_{\text{CTX-M-15}}$	0.5	0.25	0.5
E. coli	IDRL-10723	$bla_{\text{CTX-M-15}}$	0.5	0.12	0.25
E. coli	IDRL-10724	$bla_{\text{CTX-M-15}}$	0.5	1	1
E. coli	IDRL-10725	-	0.03	0.06	0.06
E. coli	IDRL-10726	$bla_{\text{CTX-M-15}}$	0.5	0.12	0.25
E. coli	IDRL-10727	$bla_{\text{CTX-M-15}}$	2	1	1
E. coli	IDRL-10728	$bla_{\text{CTX-M-15}}$	1	1	1
E. coli	IDRL-12149	$bla_{\text{OXA-48}}$	1	2	2
E. coli	IDRL-12157	$bla_{\text{OXA-48}}$	4	8	8
E. coli	IDRL-12158	bla_{NDM}	>64	>64	>64
E. coli	IDRL-12159	bla_{NDM}	8	>64	32

E. coli	IDRL-12161	*bla*$_{NDM}$	4	16	8
E. coli	IDRL-12162	*bla*$_{NDM}$	4	8	8
E. coli	IDRL-12165	*bla*$_{NDM}$	2	4	4
E. coli	IDRL-12167	*bla*$_{NDM}$	8	16	16
E. coli	IDRL-12168	*bla*$_{NDM}$	2	8	4
Klebsiella pneumoniae	IDRL-10358	*bla*$_{KPC}$	2	16	16
K. pneumoniae	IDRL-10368	*bla*$_{KPC}$	4	16	8
K. pneumoniae	IDRL-10370	*bla*$_{KPC}$	4	16	8
K. pneumoniae	IDRL-10371	*bla*$_{KPC}$	4	16	8
K. pneumoniae	IDRL-10372	*bla*$_{KPC}$	2	8	4
K. pneumoniae	IDRL-10373	*bla*$_{KPC}$	0.06	0.06	0.03
K. pneumoniae	IDRL-10374	*bla*$_{KPC}$	2	4	2
K. pneumoniae	IDRL-10376	*bla*$_{KPC}$	4	16	8
K. pneumoniae	IDRL-10377	*bla*$_{KPC}$	4	32	32
K. pneumoniae	IDRL-10378	*bla*$_{KPC}$	4	32	32
K. pneumoniae	IDRL-10379	*bla*$_{KPC}$	2	16	8
K. pneumoniae	IDRL-10380	*bla*$_{KPC}$	2	8	16
K. pneumoniae	IDRL-10381	*bla*$_{KPC}$	2	4	8
K. pneumoniae	IDRL-10382	*bla*$_{KPC}$	4	16	8
K. pneumoniae	IDRL-10383	*bla*$_{KPC}$	2	4	4
K. pneumoniae	IDRL-10384	*bla*$_{KPC}$	4	16	8
K. pneumoniae	IDRL-10385	*bla*$_{KPC}$	4	16	8
K. pneumoniae	IDRL-10386	*bla*$_{KPC}$	2	8	4
K. pneumoniae	IDRL-10387	*bla*$_{KPC}$	0.12	0.25	0.12
K. pneumoniae	IDRL-10388	*bla*$_{KPC}$	0.12	0.03	0.12
K. pneumoniae	IDRL-10390	*bla*$_{KPC}$	4	16	8
K. pneumoniae	IDRL-10391	*bla*$_{KPC}$	1	2	1
K. pneumoniae	IDRL-10394	*bla*$_{KPC}$	2	4	4
K. pneumoniae	IDRL-10395	*bla*$_{KPC}$	4	16	16
K. pneumoniae	IDRL-10396	*bla*$_{KPC}$	2	16	8
K. pneumoniae	IDRL-10397	*bla*$_{KPC}$	0.03	0.06	0.06
K. pneumoniae	IDRL-10398	*bla*$_{KPC}$	0.12	0.25	0.5
K. pneumoniae	IDRL-10400	*bla*$_{KPC}$	4	8	8
K. pneumoniae	IDRL-10401	*bla*$_{KPC}$	2	4	4
K. pneumoniae	IDRL-10408	*bla*$_{NDM}$	8	64	32
K. pneumoniae complex	IDRL-10409	*bla*$_{NDM}$	>64	>64	>64
K. pneumoniae	IDRL-10413	*bla*$_{CTX\text{-}M\text{-}12}$	0.25	1	0.5
K. pneumoniae	IDRL-10415	*bla*$_{SHV\text{-}43}$	0.06	0.06	0.06
K. pneumoniae	IDRL-10416	*bla*$_{TEM\text{-}1}$, *bla*$_{SHV\text{-}43}$	4	16	8
K. pneumoniae	IDRL-10417	*bla*$_{TEM\text{-}10,\ TEM\text{-}1}$	1	4	2
K. pneumoniae	IDRL-10422	*bla*$_{TEM\text{-}9}$	1	1	0.5
K. pneumoniae	IDRL-10425	*bla*$_{CTX\text{-}M\text{-}2}$, *bla*$_{SHV\text{-}11}$	0.06	0.12	0.12
K. pneumoniae	IDRL-10427	*bla*$_{TEM\text{-}10}$	0.5	0.25	0.5
K. pneumoniae	IDRL-10434	*bla*$_{CTX\text{-}M\text{-}15}$, *bla*$_{OXA\text{-}1}$	4	16	16
K. pneumoniae	IDRL-10437	*bla*$_{CTX\text{-}M\text{-}15}$, *bla*$_{SHV\text{-}1}$	2	8	4
K. pneumoniae complex	IDRL-10443	*bla*$_{OXA\text{-}48}$	0.25	0.12	0.25
K. pneumoniae complex	IDRL-10444	*bla*$_{OXA\text{-}48}$	0.5	0.25	0.5
K. pneumoniae	IDRL-10445	*bla*$_{IMP\text{-}1}$	1	2	2
K. pneumoniae	IDRL-10446	*bla*$_{IMP\text{-}1}$	0.25	0.25	0.25
K. pneumoniae	IDRL-10448	*bla*$_{NDM\text{-}1}$	8	64	32
K. pneumoniae	IDRL-10449	*bla*$_{IMP\text{-}1}$	0.5	1	0.5
K. pneumoniae	IDRL-10450	*bla*$_{IMP\text{-}4}$	1	2	1
K. pneumoniae	IDRL-10451	*bla*$_{NDM\text{-}1}$	4	16	16
K. pneumoniae	IDRL-10453	*bla*$_{NDM\text{-}1}$	1	2	1
K. pneumoniae	IDRL-10454	*bla*$_{NDM\text{-}1}$	1	4	2
K. pneumoniae	IDRL-10457	*bla*$_{NDM\text{-}1}$	2	4	2
K. pneumoniae	IDRL-10460	*bla*$_{IMP\text{-}1}$	4	4	8
K. pneumoniae	IDRL-10464	*bla*$_{OXA\text{-}181}$	8	64	32
K. pneumoniae	IDRL-10465	*bla*$_{NDM\text{-}1}$	4	32	32
K. pneumoniae	IDRL-10468	*bla*$_{NDM\text{-}1}$	1	4	2
K. pneumoniae	IDRL-10471	*bla*$_{NDM\text{-}1}$	1	2	1
K. pneumoniae	IDRL-10475	*bla*$_{OXA\text{-}48}$	4	8	8
K. pneumoniae	IDRL-10478	*bla*$_{OXA\text{-}232}$	1	1	0.5
K. pneumoniae	IDRL-10479	*bla*$_{OXA\text{-}48}$	0.25	0.12	0.25
K. pneumoniae	IDRL-10480	*bla*$_{OXA\text{-}232}$	0.5	1	1
K. pneumoniae	IDRL-10481	*bla*$_{OXA\text{-}48}$	0.5	0.5	0.5
K. pneumoniae	IDRL-10486	*bla*$_{NDM}$	2	4	4
K. pneumoniae	IDRL-10488	*bla*$_{NDM}$	1	2	1
K. pneumoniae	IDRL-10490	*bla*$_{NDM}$	8	64	32
K. pneumoniae	IDRL-10491	*bla*$_{OXA\text{-}48}$	0.12	0.06	0.12
K. pneumoniae	IDRL-10492	*bla*$_{OXA\text{-}48}$	0.25	0.06	0.12
K. pneumoniae	IDRL-10494	*bla*$_{OXA\text{-}48}$	1	1	0.5
K. pneumoniae	IDRL-10499	*bla*$_{KPC}$	4	8	8
K. pneumoniae	IDRL-10500	*bla*$_{KPC}$	2	4	4
K. pneumoniae	IDRL-10501	*bla*$_{KPC}$	4	16	16
K. pneumoniae	IDRL-10504	*bla*$_{KPC}$	4	16	16
K. pneumoniae	IDRL-10505	*bla*$_{KPC}$	4	16	16
K. pneumoniae	IDRL-10506	*bla*$_{NDM}$	2	2	2
K. pneumoniae	IDRL-10507	*bla*$_{KPC}$	4	16	16
K. pneumoniae	IDRL-10508	*bla*$_{KPC}$	1	2	1
K. pneumoniae	IDRL-10509	*bla*$_{KPC}$	1	1	0.5
K. pneumoniae	IDRL-10510	*bla*$_{KPC}$	2	4	4
K. pneumoniae	IDRL-10511	*bla*$_{KPC}$	4	16	16
K. pneumoniae	IDRL-10512	*bla*$_{KPC}$	0.5	0.5	0.5
K. pneumoniae	IDRL-10513	*bla*$_{KPC}$	0.5	0.5	0.5
K. pneumoniae	IDRL-10514	*bla*$_{NDM}$	1	2	1
K. pneumoniae	IDRL-10515	*bla*$_{KPC}$	1	1	0.5
K. pneumoniae	IDRL-10523	*bla*$_{KPC}$	2	8	4
K. pneumoniae	IDRL-10524	*bla*$_{KPC}$	0.5	0.5	0.5
K. pneumoniae	IDRL-10526	*bla*$_{KPC}$	4	8	8
K. pneumoniae	IDRL-10527	*bla*$_{KPC}$	4	16	16
K. pneumoniae	IDRL-10528	*bla*$_{KPC}$	4	16	16
K. pneumoniae	IDRL-10529	*bla*$_{KPC}$	1	1	0.5
K. pneumoniae	IDRL-10532	*bla*$_{KPC}$	8	8	32
K. pneumoniae	IDRL-10535	*bla*$_{KPC}$	8	32	32
K. pneumoniae	IDRL-10537	*bla*$_{KPC}$	0.5	1	0.5

K. pneumoniae	IDRL-10538	*bla*$_{\text{KPC}}$	0.25	0.25	0.25
K. pneumoniae	IDRL-10539	*bla*$_{\text{KPC}}$	8	32	32
K. pneumoniae	IDRL-10540	*bla*$_{\text{KPC}}$	4	16	16
K. pneumoniae	IDRL-10541	*bla*$_{\text{KPC}}$	4	16	16
K. pneumoniae	IDRL-10542	*bla*$_{\text{KPC}}$	0.06	0.12	0.12
K. pneumoniae	IDRL-10543	*bla*$_{\text{KPC}}$	0.12	0.12	0.25
K. pneumoniae	IDRL-10544	*bla*$_{\text{KPC}}$	2	8	8
K. pneumoniae	IDRL-10545	*bla*$_{\text{KPC}}$	4	16	16
K. pneumoniae	IDRL-10546	*bla*$_{\text{KPC}}$	0.25	0.25	0.5
K. pneumoniae	IDRL-10547	*bla*$_{\text{KPC}}$	1	1	1
K. pneumoniae	IDRL-10548	*bla*$_{\text{KPC}}$	1	1	1
K. pneumoniae	IDRL-10549	*bla*$_{\text{KPC}}$	0.25	0.12	0.25
K. pneumoniae	IDRL-10550	*bla*$_{\text{KPC}}$	0.12	0.12	0.25
K. pneumoniae	IDRL-10551	*bla*$_{\text{KPC}}$	0.12	0.25	0.25
K. pneumoniae	IDRL-10552	*bla*$_{\text{KPC}}$	0.5	0.25	0.5
K. pneumoniae	IDRL-10554	*bla*$_{\text{KPC}}$	8	32	32
K. pneumoniae	IDRL-10555	*bla*$_{\text{KPC}}$	0.5	0.5	0.5
K. pneumoniae	IDRL-10556	*bla*$_{\text{KPC}}$	0.03	0.06	0.25
K. pneumoniae	IDRL-10557	*bla*$_{\text{KPC}}$	1	2	2
K. pneumoniae	IDRL-10558	*bla*$_{\text{KPC}}$	0.12	0.03	0.25
K. pneumoniae	IDRL-10559	*bla*$_{\text{KPC}}$	4	16	16
K. pneumoniae	IDRL-10560	*bla*$_{\text{KPC}}$	4	4	8
K. pneumoniae	IDRL-10561	*bla*$_{\text{KPC}}$	1	1	1
K. pneumoniae	IDRL-10562	*bla*$_{\text{KPC}}$	0.25	0.5	0.25
K. pneumoniae	IDRL-10564	*bla*$_{\text{KPC}}$	0.5	0.25	0.5
K. pneumoniae	IDRL-10565	*bla*$_{\text{KPC}}$	0.25	0.25	0.25
K. pneumoniae	IDRL-10566	*bla*$_{\text{KPC}}$	0.5	0.25	0.5
K. pneumoniae	IDRL-10567	*bla*$_{\text{KPC}}$	0.25	0.25	0.25
K. pneumoniae	IDRL-10568	*bla*$_{\text{KPC}}$	2	2	2
K. pneumoniae	IDRL-10569	*bla*$_{\text{KPC}}$	4	16	16
K. pneumoniae	IDRL-10570	*bla*$_{\text{KPC}}$	1	4	4
K. pneumoniae	IDRL-10574	*bla*$_{\text{NDM}}$	8	64	32
K. pneumoniae	IDRL-10575	*bla*$_{\text{KPC}}$	0.25	0.25	1
K. pneumoniae	IDRL-10577	*bla*$_{\text{KPC}}$	4	16	16
K. pneumoniae	IDRL-10578	*bla*$_{\text{KPC}}$	0.12	0.25	0.12
K. pneumoniae	IDRL-10589	*bla*$_{\text{TEM}}$, *bla*$_{\text{SHV-12}}$	>64	>64	>64
K. pneumoniae	IDRL-10599	*bla*$_{\text{TEM-26}}$, *bla*$_{\text{SHV-1}}$	4	32	32
K. pneumoniae	IDRL-10600	*bla*$_{\text{TEM-1}}$, *bla*$_{\text{SHV}}$	2	4	4
K. pneumoniae	IDRL-10601	*bla*$_{\text{SHV-5/55}}$	1	1	1
K. pneumoniae	IDRL-10602	*bla*$_{\text{SHV-5/55}}$	1	1	1
K. pneumoniae	IDRL-10626	*bla*$_{\text{SHV-12}}$	1	2	2
K. pneumoniae	IDRL-10639	*bla*$_{\text{CTX-M-1}}$, *bla*$_{\text{SHV-1}}$	0.5	0.5	1
K. pneumoniae	IDRL-10647	*bla*$_{\text{CTX-M-2}}$, *bla*$_{\text{TEM-1}}$, *bla*$_{\text{SHV-11}}$	1	0.5	0.5
K. pneumoniae	IDRL-10648	*bla*$_{\text{SHV-1}}$	8	64	32
K. pneumoniae	IDRL-10652	*bla*$_{\text{TEM-26}}$, *bla*$_{\text{SHV-1}}$	1	1	2
K. pneumoniae	IDRL-10656	*bla*$_{\text{TEM-1}}$, *bla*$_{\text{SHV-12}}$	4	8	8
K. pneumoniae	IDRL-10660	*bla*$_{\text{TEM}}$, *bla*$_{\text{SHV-2}}$	1	2	2
K. pneumoniae	IDRL-10661	*bla*$_{\text{TEM-26}}$	1	2	2
K. pneumoniae	IDRL-10662	*bla*$_{\text{TEM-1}}$, *bla*$_{\text{SHV}}$	4	16	16
K. pneumoniae	IDRL-10663	*bla*$_{\text{SHV-11}}$	1	4	4
K. pneumoniae	IDRL-10664	*bla*$_{\text{TEM-6}}$, *bla*$_{\text{SHV-1}}$	0.5	0.5	1
K. pneumoniae	IDRL-10666	*bla*$_{\text{TEM-10}}$, *bla*$_{\text{SHV}}$	4	16	16
K. pneumoniae	IDRL-10667	*bla*$_{\text{TEM-1}}$, *bla*$_{\text{SHV-5/55}}$, *bla*$_{\text{CTX-M-1}}$	4	16	16
K. pneumoniae	IDRL-11412	*bla*$_{\text{OXA-48}}$	0.06	0.015	0.06
K. pneumoniae	IDRL-12147	*bla*$_{\text{OXA-48}}$	0.5	0.12	0.5
K. pneumoniae	IDRL-12148	*bla*$_{\text{OXA-48}}$	>64	>64	>64
K. pneumoniae	IDRL-12151	*bla*$_{\text{OXA-48}}$	1	2	2
K. pneumoniae	IDRL-12160	*bla*$_{\text{NDM}}$	2	1	2
K. pneumoniae	IDRL-12163	*bla*$_{\text{NDM}}$	2	4	4
K. pneumoniae	IDRL-12166	*bla*$_{\text{NDM}}$	1	1	1
K. pneumoniae	IDRL-12169	*bla*$_{\text{KPC}}$	0.5	0.25	0.5
K. pneumoniae	IDRL-12171	*bla*$_{\text{KPC}}$	2	8	8
K. pneumoniae	IDRL-12172	*bla*$_{\text{KPC}}$	0.25	0.5	0.25
K. pneumoniae	IDRL-12174	*bla*$_{\text{KPC}}$	4	16	16
K. pneumoniae	IHMA-1142926	*bla*$_{\text{SHV-OSBL;}}$ *bla*$_{\text{TEM-OSBL;}}$ *bla*$_{\text{CTX-M-1 GROUP}}$	4	16	32
K. pneumoniae	IHMA-1211353	*bla*$_{\text{SHV-OSBL;}}$ *bla*$_{\text{TEM-OSBL;}}$ *bla*$_{\text{CTX-M-15;}}$ *bla*$_{\text{KPC-2}}$	4	16	8
K. pneumoniae	IHMA-1246905	*bla*$_{\text{SHV-OSBL;}}$ *bla*$_{\text{TEM-OSBL;}}$ *bla*$_{\text{CTX-M-1 GROUP,}}$ *bla*$_{\text{NDM-1}}$	8	32	32
K. pneumoniae	IHMA-1247970	*bla*$_{\text{SHV-OSBL;}}$ *bla*$_{\text{TEM-OSBL;}}$ *bla*$_{\text{CTX-M-1 GROUP,}}$ *bla*$_{\text{NDM-1}}$	8	32	32
K. pneumoniae	IHMA-1261283	*bla*$_{\text{SHV-OSBL;}}$ *bla*$_{\text{TEM-OSBL,}}$ *bla*$_{\text{KPC-3}}$	4	16	16
K. pneumoniae	IHMA-1269211	*bla*$_{\text{SHV-OSBL;}}$ *bla*$_{\text{TEM-OSBL}}$	16	32	32
K. pneumoniae	IHMA-1273401	*bla*$_{\text{SHV-12;}}$ *bla*$_{\text{KPC-2}}$	8	32	32
K. pneumoniae	IHMA-1294760	*bla*$_{\text{SHV-12;}}$ *bla*$_{\text{TEM-OSBL;}}$ *bla*$_{\text{KPC-2}}$	4	8	8
K. pneumoniae	IHMA-1296510	*bla*$_{\text{SHV-12;}}$ *bla*$_{\text{TEM-OSBL;}}$ *bla*$_{\text{KPC-3}}$	8	8	32
K. pneumoniae	IHMA-1319427	*bla*$_{\text{SHV-12;}}$ *bla*$_{\text{TEM-OSBL}}$	32	32	64
K. pneumoniae	IHMA-1369251	*bla*$_{\text{SHV-12;}}$ *bla*$_{\text{TEM-OSBL;}}$ *bla*$_{\text{NDM-6}}$	8	16	32
Enterobacter cloacae	IDRL-10375	*bla*$_{\text{KPC}}$	0.5	0.5	0.25
E. cloacae	IDRL-10392	*bla*$_{\text{KPC}}$	0.25	0.25	0.25
E. cloacae	IDRL-10399	*bla*$_{\text{KPC}}$	0.5	0.25	0.25
E. cloacae	IDRL-10403	*bla*$_{\text{KPC}}$	2	2	4
E. cloacae	IDRL-10405	*bla*$_{\text{KPC}}$	8	16	4
E. cloacae	IDRL-10459	*bla*$_{\text{IMP-1}}$	2	2	2
E. cloacae	IDRL-10461	*bla*$_{\text{IMP-1}}$	16	32	32
E. cloacae	IDRL-10462	*bla*$_{\text{NDM-1}}$	4	8	8
E. cloacae	IDRL-10463	*bla*$_{\text{NDM-1}}$	8	64	16
E. cloacae	IDRL-10470	*bla*$_{\text{NDM-1}}$	1	1	4
E. cloacae	IDRL-10474	*bla*$_{\text{NDM-1}}$	4	8	8
E. cloacae	IDRL-10476	*bla*$_{\text{IMI-1}}$	1	2	2
E. cloacae	IDRL-10477	*bla*$_{\text{IMI-1}}$	0.5	0.5	0.06
E. cloacae complex	IDRL-10484	*bla*$_{\text{IMP}}$	16	32	32
E. cloacae complex	IDRL-10485	*bla*$_{\text{IMP}}$	2	8	2
E. cloacae complex	IDRL-10487	*bla*$_{\text{NDM}}$	2	4	2
E. cloacae complex	IDRL-10496	*bla*$_{\text{IMI}}$	1	1	1
E. cloacae complex	IDRL-10497	*bla*$_{\text{IMI-1}}$	0.5	1	1
E. cloacae complex	IDRL-10498	*bla*$_{\text{IMI-1}}$	0.5	1	1
E. cloacae	IDRL-10517	*bla*$_{\text{KPC}}$	0.25	0.5	2
E. cloacae complex	IDRL-10631	*bla*$_{\text{CTX-M-9}}$	0.25	0.12	0.06

E. cloacae	IHMA-1100868	$bla_{\text{TEM-OSBL}}$; $bla_{\text{CTX-M-15}}$; $bla_{\text{KPC-2}}$	32	64	64
E. cloacae	IHMA-1114332	$bla_{\text{NDM-7}}$	32	>64	16
E. cloacae	IHMA-1178662	$bla_{\text{TEM-OSBL}}$; $bla_{\text{CTX-M-15}}$; $bla_{\text{ACT-TYPE}}$; $bla_{\text{NDM-1}}$	2	2	4
E. cloacae	IHMA-1207294	$bla_{\text{SHV-12}}$; $bla_{\text{TEM-OSBL}}$; $bla_{\text{CTX-M-15}}$; $bla_{\text{ACT-TYPE}}$; $bla_{\text{NDM-1}}$	8	16	32
E. cloacae	IHMA-1207298	$bla_{\text{SHV-12}}$; $bla_{\text{TEM-OSBL}}$; $bla_{\text{CTX-M-15}}$; $bla_{\text{ACT-TYPE}}$; $bla_{\text{NDM-1}}$	8	16	8
E. cloacae	IHMA-1207309	$bla_{\text{SHV-12}}$; $bla_{\text{TEM-OSBL}}$; $bla_{\text{CTX-M-15}}$; $bla_{\text{ACT-TYPE}}$; $bla_{\text{NDM-1}}$	8	8	16
E. cloacae	IHMA-1207332	$bla_{\text{SHV-12}}$; $bla_{\text{TEM-OSBL}}$; $bla_{\text{CTX-M-15}}$; $bla_{\text{ACT-TYPE}}$; $bla_{\text{NDM-1}}$	4	4	8
E. cloacae	IHMA-1207515	$bla_{\text{SHV-12}}$; $bla_{\text{TEM-OSBL}}$; $bla_{\text{CTX-M-15}}$; $bla_{\text{ACT-TYPE; NDM-6}}$	8	32	32
E. cloacae	IHMA-1212160	$bla_{\text{SHV-12; CTX-M-9}}$; $bla_{\text{ACT-TYPE}}$; $bla_{\text{NDM-6}}$	16	32	32
E. cloacae	IHMA-1242680	$bla_{\text{CTX-M-15}}$; $bla_{\text{ACT-TYPE}}$; $bla_{\text{NDM-1}}$	8	16	16
E. cloacae	IHMA-1249054	$bla_{\text{VIM-1}}$	2	4	4
E. cloacae	IHMA-1270823	$bla_{\text{NDM-1}}$	32	32	32
E. cloacae	IHMA-1277450	$bla_{\text{SHV-12}}$; $bla_{\text{NDM-1}}$	8	32	32
E. cloacae	IHMA-1277478	$bla_{\text{SHV-12}}$; $bla_{\text{NDM-1}}$	8	32	32
E. cloacae	IHMA-1324577	$bla_{\text{SHV-12}}$; $bla_{\text{TEM-OSBL}}$; $bla_{\text{CTX-M-15}}$; $bla_{\text{NDM-1}}$	8	8	16
Klebsiella aerogenes	IDRL-10369	bla_{KPC}	4	8	8
K. aerogenes	IDRL-10404	bla_{KPC}	4	16	16
K. aerogenes	IDRL-10536	bla_{KPC}	0.25	0.25	4
K. aerogenes	IHMA-1211388	$bla_{\text{TEM-OSBL}}$; $bla_{\text{KPC-2}}$	8	16	32
K. aerogenes	IHMA-1211454	$bla_{\text{TEM-OSBL}}$; $bla_{\text{KPC-2}}$	8	16	64
K. aerogenes	IHMA-1211469	$bla_{\text{TEM-OSBL}}$; $bla_{\text{KPC-2}}$	8	32	32
K. aerogenes	IHMA-1252218	-	4	8	8
Morganella morgannii	IDRL-10675	$bla_{\text{CMY-2}}$	0.12	0.25	0.25
M. morgannii	IDRL-10676	$bla_{\text{CMY-2}}$	0.12	0.25	0.25
Serratia marcescens	IDRL-10393	bla_{KPC}	64	64	64
S. marcescens	IDRL-10410	bla_{SME}	0.03	0.015	0.12
S. marcescens	IDRL-10442	bla_{SME}	0.12	0.03	0.06
S. marcescens	IDRL-10502	bla_{SME}	0.25	1	0.25
S. marcescens	IDRL-10519	bla_{KPC}	2	4	8
S. marcescens	IHMA-1230547	$bla_{\text{TEM-10}}$	32	32	64
S. marcescens	IHMA-1233240	$bla_{\text{SHV-12}}$	4	8	16
Citrobacter freundii	IDRL-10367	bla_{KPC}	2	8	4
C. freundii	IDRL-10472	$bla_{\text{IMP-1}}$	2	4	4
C. freundii	IDRL-10489	bla_{NDM}	2	16	8
C. freundii	IDRL-10518	bla_{KPC}	0.12	0.25	0.5
C. freundii	IDRL-12173	bla_{KPC}	0.5	0.12	0.03
C. freundii	IDRL-12175	bla_{KPC}	2	4	1
C. freundii	IDRL-12177	bla_{KPC}	1	1	0.25
C. freundii	IDRL-12178	bla_{KPC}	0.25	0.12	0.06
C. freundii	IHMA-1107488	$bla_{\text{SHV-12}}$; $bla_{\text{CMY-TYPE}}$; $bla_{\text{VIM-1}}$	4	4	4
Citrobacter koseri	IDRL-10428	bla_{KPC}	8	16	8
C. koseri	IDRL-10493	$bla_{\text{OXA-48}}$	0.25	0.25	0.5
C. koseri	IDRL-10533	bla_{KPC}	1	1	0.5
C. koseri	IDRL-12153	bla_{OXA}	1	1	0.25
Providencia stuartii	IDRL-10389	bla_{KPC}	0.03	0.25	0.06
P. stuartii	IDRL-10576	bla_{KPC}	0.03	0.06	0.12
Klebsiella oxytoca	IDRL-12170	bla_{KPC}	4	8	8
Proteus mirabilis	IDRL-10406	bla_{KPC}	0.015	0.06	0.25
Critobacter sedlakii	IDRL-10473	$bla_{\text{NDM-1}}$	1	2	4
Pseudomonas aeruginosa	IDRL-10412	-	1	2	1
P. aeruginosa	IDRL-10530	-	0.25	0.5	1
P. aeruginosa	IDRL-10534	-	0.5	2	2
P. aeruginosa	IDRL-10563	-	0.5	1	2
P. aeruginosa	IDRL-10628	-	1	2	1
P. aeruginosa	IDRL-11622	-	0.12	0.12	0.25
P. aeruginosa	IDRL-11650	-	1	2	2
P. aeruginosa	IDRL-11660	-	2	8	4
P. aeruginosa	IDRL-11686	-	0.5	4	4
P. aeruginosa	IDRL-11688	-	0.5	2	0.5
P. aeruginosa	IDRL-11690	-	0.5	0.5	0.5
P. aeruginosa	IDRL-11691	-	0.12	0.12	0.12
P. aeruginosa	IDRL-11696	-	0.5	0.5	1
P. aeruginosa	IDRL-11699	-	1	2	1
P. aeruginosa	IDRL-11701	-	0.25	0.5	2
P. aeruginosa	IDRL-11704	-	0.5	2	1
P. aeruginosa	IDRL-11706	-	1	2	1
P. aeruginosa	IDRL-11707	-	1	2	2
P. aeruginosa	IDRL-11720	-	0.06	0.12	0.5
P. aeruginosa	IDRL-11723	-	0.5	1	1
P. aeruginosa	IDRL-11736	-	0.25	0.5	1
P. aeruginosa	IDRL-11737	-	0.12	0.25	0.5
P. aeruginosa	IDRL-11738	-	0.25	1	2
P. aeruginosa	IDRL-11739	-	0.25	0.5	1
P. aeruginosa	IDRL-11740	-	0.25	1	0.5
P. aeruginosa	IDRL-11741	-	0.25	0.5	1
P. aeruginosa	IDRL-11742	-	0.5	1	2
P. aeruginosa	IDRL-11743	-	2	8	4
P. aeruginosa	IDRL-11744	-	0.12	0.5	0.5
P. aeruginosa	IDRL-11745	-	0.25	2	1
P. aeruginosa	IDRL-11746	-	2	4	8
P. aeruginosa	IDRL-11747	-	0.25	2	1
P. aeruginosa	IDRL-11748	-	0.25	1	0.5
P. aeruginosa	IDRL-11749	-	0.25	1	1
P. aeruginosa	IDRL-11750	-	1	2	4
P. aeruginosa	IDRL-11751	-	0.25	1	0.5
P. aeruginosa	IDRL-11752	-	0.25	0.5	0.5
P. aeruginosa	IDRL-11753	-	0.25	2	2
P. aeruginosa	IDRL-11754	-	0.5	1	1
P. aeruginosa	IDRL-11755	-	0.06	0.5	0.5
P. aeruginosa	IDRL-11756	-	0.5	2	4
P. aeruginosa	IDRL-11757	-	0.12	0.5	2
P. aeruginosa	IDRL-11758	-	0.5	4	8
P. aeruginosa	IDRL-11760	-	0.5	4	8
P. aeruginosa	IDRL-11761	-	0.5	1	8
P. aeruginosa	IDRL-11762	-	0.25	1	8
P. aeruginosa	IDRL-11763	-	0.5	1	2

P. aeruginosa	IDRL-11764	-	1	2	8
P. aeruginosa	IDRL-11765	-	1	2	4
P. aeruginosa	IDRL-11766	-	1	4	8
P. aeruginosa	IDRL-11767	-	0.25	1	2
P. aeruginosa	IDRL-11768	-	1	1	8
P. aeruginosa	IDRL-11769	-	0.008	0.015	0.06
P. aeruginosa	IDRL-11789	-	0.25	0.5	4
P. aeruginosa	IDRL-12185	-	0.5	1	2
P. aeruginosa	IHMA-1205534	-	2	2	2
P. aeruginosa	IHMA-1231459	-	16	16	16
P. aeruginosa	IHMA-1276161	*bla*$_{\text{PER-1}}$	32	32	32
Stenotrophomonas maltophilia	IDRL-5070	-	0.25	0.25	1
S. maltophilia	IDRL-5071	-	0.008	0.015	0.25
S. maltophilia	IDRL-5072	-	0.03	0.06	0.12
S. maltophilia	IDRL-5073	-	0.06	0.06	0.25
S. maltophilia	IDRL-5075	-	0.5	1	2
S. maltophilia	IDRL-5076	-	0.03	0.03	0.12
S. maltophilia	IDRL-5077	-	0.03	0.06	0.25
S. maltophilia	IDRL-5078	-	0.06	0.06	0.25
S. maltophilia	IDRL-5079	-	0.06	0.06	0.06
S. maltophilia	IDRL-5080	-	0.06	0.06	0.5
S. maltophilia	IDRL-5081	-	0.015	0.03	0.12
S. maltophilia	IDRL-5082	-	0.004	0.008	0.12
S. maltophilia	IDRL-5084	-	0.06	0.06	0.5
S. maltophilia	IDRL-5085	-	0.12	0.06	0.5
S. maltophilia	IDRL-5086	-	0.015	0.03	0.06
S. maltophilia	IDRL-5087	-	0.06	0.06	1
S. maltophilia	IDRL-5088	-	0.12	0.25	1
S. maltophilia	IDRL-5089	-	0.03	0.03	0.25
S. maltophilia	IDRL-5090	-	0.12	0.25	1
S. maltophilia	IDRL-5091	-	0.015	0.03	0.12
S. maltophilia	IDRL-5092	-	0.06	0.12	1
S. maltophilia	IDRL-5093	-	0.015	0.03	0.25
S. maltophilia	IDRL-5094	-	0.015	0.03	0.06
S. maltophilia	IDRL-5096	-	0.008	0.015	0.06
S. maltophilia	IDRL-5097	-	0.015	0.015	0.12
S. maltophilia	IDRL-5128	-	0.015	0.03	0.03
S. maltophilia	IDRL-5129	-	0.12	0.12	0.25
S. maltophilia	IDRL-5131	-	0.03	0.12	0.03
S. maltophilia	IDRL-5132	-	0.06	0.03	0.06
S. maltophilia	IDRL-5133	-	0.06	0.06	0.03
S. maltophilia	IDRL-5134	-	0.12	0.06	0.25
S. maltophilia	IDRL-5135	-	0.06	0.06	0.03
S. maltophilia	IDRL-5136	-	0.25	0.12	0.12
S. maltophilia	IDRL-5137	-	0.25	0.25	0.12
S. maltophilia	IDRL-5138	-	0.25	0.25	0.12
S. maltophilia	IDRL-5139	-	0.12	0.12	0.25
S. maltophilia	IDRL-5142	-	0.015	0.015	0.12
S. maltophilia	IDRL-5143	-	0.015	0.03	0.03
S. maltophilia	IDRL-5145	-	0.015	0.03	0.12
S. maltophilia	IDRL-5146	-	0.06	0.06	0.03
S. maltophilia	IDRL-5148	-	0.03	0.06	0.06
S. maltophilia	IDRL-5149	-	0.015	0.03	0.25
S. maltophilia	IDRL-5150	-	0.12	0.25	0.25
S. maltophilia	IDRL-5152	-	0.015	0.03	0.03
S. maltophilia	IDRL-5153	-	0.06	0.12	0.25
S. maltophilia	IDRL-5154	-	0.015	0.03	0.06
S. maltophilia	IDRL-5155	-	0.12	0.25	0.03
S. maltophilia	IDRL-5156	-	0.06	0.12	0.25
S. maltophilia	IDRL-5157	-	0.015	0.03	1
S. maltophilia	IDRL-5158	-	0.03	0.12	0.5
S. maltophilia	IDRL-5160	-	0.03	0.06	0.25
S. maltophilia	IDRL-5161	-	0.12	0.25	0.5
S. maltophilia	IDRL-5162	-	0.25	0.5	1
S. maltophilia	IDRL-5163	-	0.25	0.5	1
S. maltophilia	IDRL-5164	-	0.06	0.25	1
S. maltophilia	IDRL-5165	-	0.015	0.03	0.12
S. maltophilia	IDRL-5166	-	0.03	0.12	1
S. maltophilia	IDRL-5168	-	0.03	0.06	0.06
S. maltophilia	IDRL-5169	-	0.25	0.5	1
S. maltophilia	IDRL-5170	-	0.25	0.25	1
S. maltophilia	IDRL-5171	-	0.015	0.03	0.12
S. maltophilia	IDRL-5172	-	0.12	0.12	1
S. maltophilia	IDRL-5173	-	0.25	0.25	1
S. maltophilia	IDRL-5174	-	0.25	0.5	4
S. maltophilia	IDRL-5175	-	0.06	0.06	1
S. maltophilia	IDRL-5226	-	0.25	0.5	0.5
S. maltophilia	IDRL-5227	-	0.25	1	1
S. maltophilia	IDRL-5228	-	0.06	0.12	0.5
S. maltophilia	IDRL-7117	-	0.12	0.25	0.25
S. maltophilia	IDRL-7315	-	0.25	0.5	1
S. maltophilia	IDRL-8309	-	0.12	0.12	0.25
Acinetobacter baumannii	IDRL-9919	-	0.06	0.12	0.25
A. baumannii	IDRL-11626	-	0.12	0.25	2
A. baumannii	IDRL-11629	-	8	16	16
A. baumannii	IDRL-11631	-	0.03	0.06	1
A. baumannii	IDRL-11632	-	0.25	0.25	2
A. baumannii	IDRL-11641	-	0.12	0.25	2
A. baumannii	IHMA-1121211	*bla*$_{\text{PER-1;}}$ *bla*$_{\text{OXA-23}}$	64	32	>64
A. baumannii	IHMA-1121896	*bla*$_{\text{PER-1;}}$ *bla*$_{\text{OXA-72}}$	16	>64	>64
A. baumannii	IHMA-1121897	*bla*$_{\text{PER-1;}}$ *bla*$_{\text{OXA-72}}$	4	8	64
A. baumannii	IHMA-1121900	*bla*$_{\text{PER-1;}}$ *bla*$_{\text{OXA-72}}$	8	8	64
A. baumannii	IHMA-1122558	*bla*$_{\text{TEM-OSBL;}}$ *bla*$_{\text{PER-1}}$	16	32	64
A. baumannii	IHMA-1215489	-	4	4	32
A. baumannii	IHMA-1247945	*bla*$_{\text{PER-1;}}$ *bla*$_{\text{OXA-23-TYPE}}$	16	32	8
A. baumannii	IHMA-1264808	-	2	2	8

A. baumannii	IHMA-1266661	$bla_{\text{OXA-24-TYPE}}$	4	4	16
A. baumannii	IHMA-1266738	$bla_{\text{PER-1}}$; $bla_{\text{OXA-24-TYPE}}$	32	32	32
A. baumannii	IHMA-1470727	$bla_{\text{PER-7}}$; $bla_{\text{NDM-1}}$; $bla_{\text{OXA-23-TYPE}}$	8	32	64
A. baumannii	IHMA-1908951	-	0.5	1	8
A. baumannii	IHMA-1916787	-	>64	>64	>64
A. baumannii	IHMA-1939212	-	0.5	1	8
A. baumannii	IHMA-1939213	-	0.03	0.25	1
A. baumannii	IHMA-1939214	-	64	64	16
A. baumannii	IHMA-1939215	-	0.06	0.12	2
A. baumannii	IHMA-1939216	-	0.06	0.12	2
A. baumannii	IHMA-1939218	-	0.06	0.12	0.25
A. baumannii	IHMA-1939205	-	0.25	0.5	1
A. baumannii	IHMA-1939206	-	0.06	0.12	1
A. baumannii	IHMA-1939207	-	0.12	0.25	1
A. baumannii	IHMA-1939208	-	0.12	0.25	1
A. baumannii	IHMA-1939209	-	0.25	0.12	1
A. baumannii	IHMA-1939210	-	0.06	0.25	0.12
A. baumannii	IHMA-1939217	-	0.25	0.5	2
A. baumannii	IHMA-1939829	-	0.12	0.25	1
A. baumannii	IHMA-1939833	-	0.12	0.5	2
A. baumannii	IHMA-1939836	-	0.12	0.25	1
A. baumannii	IHMA-1939839	-	0.06	0.12	1
A. baumannii	IHMA-1557389	-	0.06	0.12	0.25
A. baumannii	IHMA-1939837	-	0.12	0.25	0.5
A. baumannii	IHMA-1935985	-	0.06	0.25	1
A. baumannii	IHMA-1935986	-	0.06	0.12	0.5
A. baumannii	IHMA-1935989	-	0.12	0.25	1
A. baumannii	IHMA-1935988	-	0.03	0.06	0.12
A. baumannii	IHMA-1909814	-	16	16	64
A. baumannii	IHMA-1909886	-	16	16	32
A. baumannii	IHMA-1721239	-	>64	>64	32
A. baumannii	IHMA-1935592	-	0.06	0.06	1
A. baumannii	IHMA-1935593	-	0.12	0.25	1
A. baumannii	IHMA-1935594	-	0.5	0.5	4
A. baumannii	IHMA-1935595	-	0.12	0.25	2
A. baumannii	IHMA-1935596	-	0.03	0.06	0.5
A. baumannii	IHMA-1935597	-	0.25	0.5	2
A. baumannii	IHMA-1935599	-	0.06	0.12	2
A. baumannii	IHMA-1935601	-	0.03	0.06	2
A. baumannii	IHMA-1935602	-	0.06	0.12	2
A. baumannii	IHMA-1916476	-	16	32	8
A. baumannii	IHMA-1932840	-	0.03	0.06	0.5
A. baumannii	IHMA-1721345	-	8	16	64
A. baumannii	IHMA-1721555	-	64	>64	32
A. baumannii	IHMA-1721578	-	16	64	16
A. baumannii	IHMA-1922835	-	16	32	16
A. baumannii	IHMA-1922836	-	32	64	64
A. baumannii	IHMA-1922837	-	16	32	16
A. baumannii	IHMA-1922839	-	32	32	32
A. baumannii	IHMA-1922843	-	>64	>64	32
A. baumannii	IHMA-1922842	-	0.06	0.25	2
A. baumannii	IHMA-1721728	-	16	32	16
A. baumannii	IHMA-1922871	-	16	32	16
A. baumannii	IHMA-1923238	-	>64	>64	16
A. baumannii	IHMA-1923239	-	32	>64	64
A. baumannii	IHMA-1923309	-	>64	>64	>64
A. baumannii	IHMA-1923304	-	32	>64	>64
A. baumannii	IHMA-1923570	-	32	32	32
A. baumannii	IHMA-1923636	-	32	64	64
A. baumannii	IHMA-1923637	-	>64	>64	>64
A. baumannii	IHMA-1923638	-	8	32	16
A. baumannii	IHMA-1923034	-	8	32	>64
A. baumannii	IHMA-1923035	-	4	4	16
A. baumannii	IHMA-1923037	-	16	32	16
A. baumannii	IHMA-1923038	-	8	16	32
A. baumannii	IHMA-1923040	-	4	8	16
A. baumannii	IHMA-1923041	-	4	8	32
A. baumannii	IHMA-1923429	-	32	64	8
A. baumannii	IHMA-1923431	-	32	64	32
A. baumannii	IHMA-1923432	-	8	16	16
A. baumannii	IHMA-1923270	-	4	8	8
A. baumannii	IHMA-1923102	-	16	16	64
A. baumannii	IHMA-1721648	-	8	16	16
A. baumannii	IHMA-1922937	-	8	16	16
A. baumannii	IHMA-1722147	-	16	32	16
A. baumannii	IHMA-1944645	-	0.03	0.06	1
A. baumannii	IHMA-1944646	-	2	4	8
A. baumannii	IHMA-1947724	-	0.06	0.12	0.5
A. baumannii	IHMA-1947725	-	0.06	0.12	1
A. baumannii	IHMA-1947726	-	0.03	0.06	1
A. baumannii	IHMA-1947744	-	0.03	0.12	1
A. baumannii	IHMA-1521197	-	0.12	0.25	2
A. baumannii	IHMA-1939308	-	0.015	0.03	0.25
Burkholderia cepacia complex	IHMA-1219352	-	8	16	8
B. vietnamiensis	IDRL-12145	-	0.015	0.03	0.015
B. ambifaria	IDRL-12146	-	0.015	0.03	0.03
B. cenocepacia	IDRL-12186	-	1	1	0.06
B. multivorans	IDRL-12187	-	0.03	0.03	0.06
B. multivorans	IDRL-12188	-	0.015	0.03	0.03
B. multivorans	IDRL-12189	-	0.015	0.03	0.03
B. multivorans	IDRL-12190	-	16	32	32
B. cenocepacia	IDRL-12191	-	0.008	0.03	0.03
B. multivorans	IDRL-12192	-	0.008	0.015	0.03
B. cepacia complex	IDRL-12194	-	0.03	0.03	0.06
B. cepacia complex	IDRL-12211	-	0.008	0.015	0.03
B. multivorans	IDRL-12212	-	8	16	8

B. cepacia complex	IDRL-12213	-	8	16	8
B. cepacia complex	IDRL-12214	-	0.008	0.008	0.015
B. vietnamiensis	IDRL-12215	-	0.015	0.03	0.015
B. cenocepacia	IDRL-12216	-	0.03	0.03	0.06
B. multivorans	IDRL-12217	-	0.03	0.03	0.03
B. multivorans	IDRL-12218	-	0.015	0.015	0.03
B. cepacia complex	IDRL-12219	-	0.008	0.015	0.015
B. cepacia complex	IDRL-12220	-	0.03	0.06	0.06
B. multivorans	IDRL-12221	-	0.03	0.03	0.06
B. vietnamiensis	IDRL-12222	-	0.03	0.06	0.015
B. multivorans	IDRL-12223	-	0.015	0.015	0.06
B. cepacia complex	IDRL-12224	-	0.008	0.015	0.015
B. cepacia complex	IDRL-12225	-	0.03	0.03	0.03
B. cepacia complex	IDRL-12226	-	0.015	0.015	0.03
B. cepacia complex	IDRL-12227	-	0.06	0.12	0.06
B. multivorans	IDRL-12228	-	0.03	0.03	0.06
B. vietnamiensis	IDRL-12229	-	0.25	2	0.06
B. cenocepacia	IDRL-12230	-	0.5	1	0.25
B. cenocepacia	IDRL-12231	-	1	2	2
B. cepacia complex	IDRL-12232	-	0.008	0.015	0.015
B. vietnamiensis	IDRL-12233	-	0.03	0.06	0.12
B. cepacia complex	IDRL-12234	-	0.03	0.03	0.06
B. multivorans	IDRL-12235	-	0.015	0.015	0.06
B. cepacia complex	IDRL-12236	-	0.06	0.06	0.06
B. cepacia complex	IDRL-12237	-	0.03	0.03	0.06
B. multivorans	IDRL-12238	-	>64	>64	64
B. arboris	IDRL-12239	-	0.06	0.06	0.06
B. multivorans	IDRL-12240	-	0.015	0.03	0.06
B. multivorans	IDRL-12241	-	0.06	0.06	0.06
B. cepacia complex	IDRL-12242	-	0.015	0.015	0.06
B. arboris	IDRL-12243	-	0.03	0.06	0.06
B. latens	IDRL-12244	-	0.12	0.12	0.12
B. multivorans	IDRL-12245	-	0.03	0.06	0.06
B. multivorans	IDRL-12246	-	0.015	0.015	0.03
B. cepacia complex	IDRL-12247	-	0.015	0.03	0.015
B. cepacia complex	IDRL-12248	-	0.008	0.015	0.25

Table S2. MIC for each quality control strain and testing method.

***Pseudomonas aeruginosa* ATCC 27853**		***Escherichia coli* ATCC 25922**	
**ID-BMD**[1] (µg/mL)	**AD**[2] (µg/mL)	**ID-BMD** (µg/mL)	**AD** (µg/mL)
0.5	0.5	0.25	0.125
0.25	2	0.06	0.125
0.25	1	0.25	0.125
0.5	2	0.25	0.5
0.5	1	0.25	0.5
0.12	2	0.25	0.5
0.5	2	0.25	0.5
0.5	2	0.25	0.25
0.5	2	0.25	0.25
0.25	2	0.125	0.125
0.25	2	0.25	0.25
0.25	2	0.25	0.125
0.25	2	0.125	0.125
0.5	2	0.125	0.06
0.25	2	0.25	0.125
0.5	4	0.25	0.125
0.125	2	0.25	0.5
0.125	2	0.25	0.5
0.25	2	0.25	0.06
0.25	0.25	0.125	0.06
0.125	0.5	0.25	0.06
0.125	2	0.125	0.25
0.5	2	0.25	0.06
0.25	2	0.25	0.125
0.25	1	0.5	0.125
0.25	2	0.25	0.125
0.25	2	0.25	0.06
0.25	1	0.25	0.06
0.25	1	0.25	0.06

[1]ID-BMD: iron-depleted broth microdilution
[2]AD: agar dilution

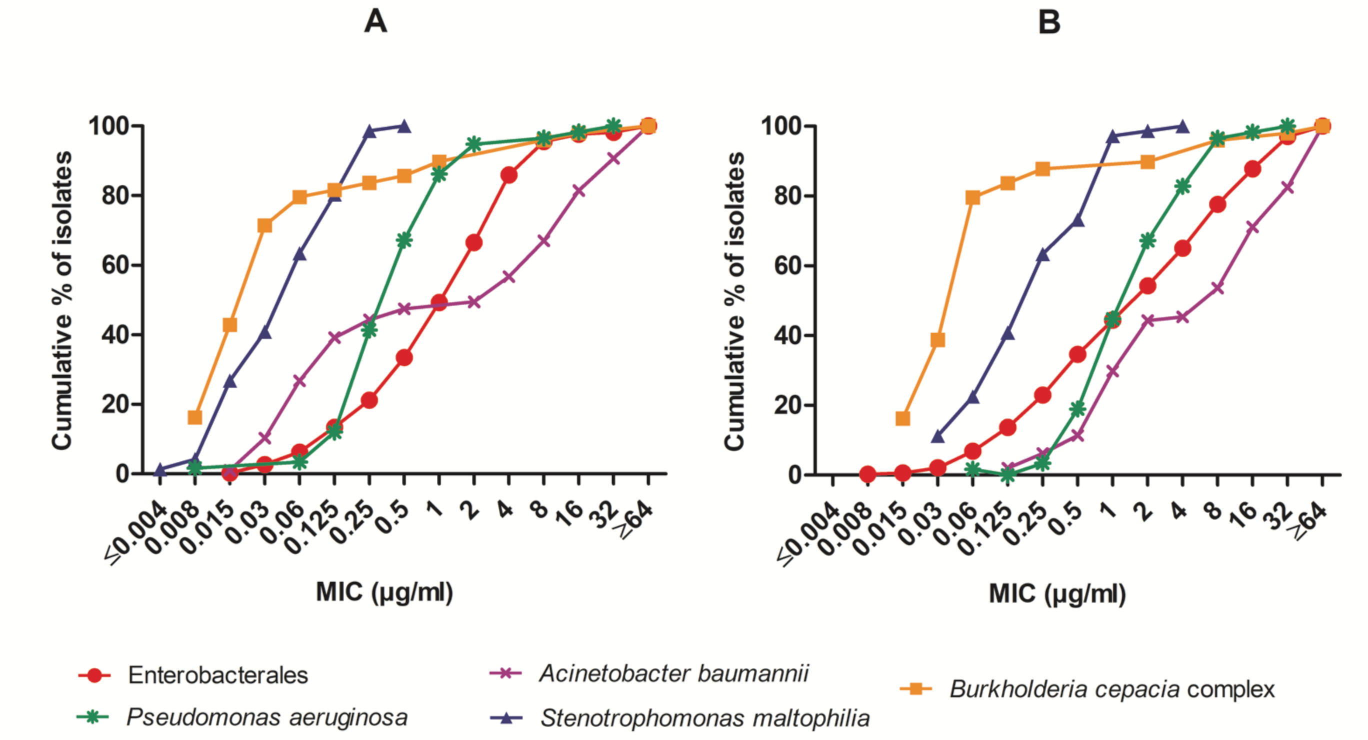

Figure S1. Cumulative percentages of isolates inhibited at specified concentrations in µg/mL for broth microdilution using iron depleted cation-adjusted Muller Hinton broth (ID-BMD) (A), and agar dilution (B).
